# Association between morbidity of non-communicable disease and employment status: a comparison between Korea and the United States

**DOI:** 10.1186/s12889-020-08883-3

**Published:** 2020-05-24

**Authors:** Sung Hee Kwon, Jun-Pyo Myong, Hyoung-Ah Kim, Kyeong Yeon Kim

**Affiliations:** 1 Department of Occupational & Environmental Medicine, Seoul St. Mary’s Hospital, College of Medicine, The Catholic University of Korea, 222, Banpo-daero, Seocho-gu, Seoul, 06591 Republic of Korea; 2 Department of Preventive Medicine, College of Medicine, The Catholic University of Korea, Seoul, Republic of Korea; 3 Korea Medical Institute, Seoul, Republic of Korea

**Keywords:** Employment status, Korea, USA, Chronic disease

## Abstract

**Background:**

Globally, the prevalence of chronic disease continues to rise and is likely to grow further over the coming decades due to population ageing. Since older age is associated closely with development of chronic disease, it stands to reason that demographic changes will increase the proportion of older workers with chronic disease. The aim of the present study was to determine how chronic diseases affect employment status in Korea and the USA.

**Methods:**

The study was based on National Health and Nutrition Survey data (2007–2014) obtained by the Korean and American Centers for Disease Control and Prevention. A total of 44,693 subjects were categorized into two geographical groups: Korea (29,260 subjects) and the USA (15,433 subjects). A *chi-square* test was used to compare the groups in terms of socio-demographic factors, health-related factors, and chronic disease. Multivariate logistic regression analysis was conducted to determine the effect of five chronic diseases (hypertension, diabetes, dyslipidemia, cardiovascular disease, and cancer) on employment status.

**Results:**

There were 29,260 Korean and 15,433 American respondents. Chronic disease increased the risk of unemployment in Korea markedly (Odds ratio [OR] range, 1.17–2.47). Cardiovascular disease and cancer had the most profound negative effect on Korean unemployment (OR = 2.47 and 2.03, respectively). The risk of unemployment was generally 2–3-fold lower in the USA (OR range, 0.5–1.04).

**Conclusions:**

Chronic disease had a significant impact on economic activity in Korea, but a smaller impact in the USA. This difference may be related to different health insurance schemes and cultural approaches to people with diseases in the two countries. It is important to explore factors that limit economic participation by people with chronic diseases, and to identify social policies that will overcome these factors. Further between-country studies are needed to identify social solutions to the socio-economic burden of chronic illness.

## Background

Chronic diseases, also known as non-communicable diseases (NCDs), are a main cause of death globally. They also associate with a significant socio-economic burden since they account for most of the governmental health care expenditure on treatment and prevention. The prevalence of chronic disease continues to rise around the world [1–3], and is likely to grow further over the coming decades due to population ageing. Since an older age associates closely with the development of chronic disease, these demographic changes will increase the proportion of older workers in the labor force who have chronic disease [4]. The resulting socio-economic impact may be significant. This explains why the labor market focuses on not only the working age but also chronically ill workers [5].

Chronic diseases directly or indirectly affect the labor market via various mechanisms. For example, chronic disease limits participation in the labor market, either by increasing the need for early retirement or by reducing the productivity of the worker [6]. These problems are particularly acute for people with multiple chronic diseases: compared with workers with no or only one chronic diseases in the United States of America (USA), people with multiple chronic diseases are both less likely to be employed and likely to miss more work days due to illness [7]. Similarly, in Europe, where 23.5% of the working population has chronic diseases, the employment rate for these people is much lower than that of those who lack a disease [8].

Countries differ markedly in terms of their labor environment, their health care systems and national economic indicators such as Gross Domestic Product and Gross National Income. These factors markedly influence the participation of workers with chronic illness in the work force. For example, countries such as the USA, which has a highly flexible labor market, provide an environment that helps people with chronic diseases to continue working [9]. The availability of health insurance also greatly impacts the labor market. Thus, for example, in countries like the USA where employment status dictates the availability and nature of health insurance, people with chronic diseases may be more inclined to stay with or seek companies that provide health insurance than people without such diseases [10]. By contrast, these factors may play a smaller role in countries like Korea that have a universal healthcare system [11]. National socio-economic indicators (such as employment) and health status shape the development of chronic diseases and the economic participation of chronically ill people [12]. Non-communicable disease, for example dyslipidemia requires adequate lifestyle measures such as diet control and exercise to achieve satisfactory control [13, 14]. The aim of the present study was to determine how chronic diseases affect employment status in Korea and the USA. For this, data from the National Health and Nutrition Examination Survey (NHANES) carried out in Korea and the USA were analyzed. These national data are comparable because the surveys in both countries were conducted according to World Health Organization standards and thus employed the same data sources and methods [15].

## Methods

### Study setting and participants

This study was based on data from the annual or biennial NHANES conducted in Korea (called KNHANES) and in the USA (called NHANES) by their respective Centers of Disease Control and Prevention between 2007 and 2014. The NHANES sample is designed to be nationally representative of the civilian, non-institutionalized U.S. population and each year’s sample and any combination of samples from consecutive years comprised a nationally representative sample of then resident, non-institutionalized U.S. population [15]. The KNHANES is designed following NHANES in USA as a complex, stratified, multistage probability-cluster survey of a representative sample of the non-institutionalized civilian population in Korea. In both surveys, participants from representative areas of each nation were selected by random sampling and asked (in interviews) about their health, health-related behavior, and nutrition. The interview response rate by KNHANES in fourth, fifth and sixth waves were 78.4, 80.8, and 78.3%, respectively. For NHANES, the response rate in period ‘2007–2008’, ‘2009–2010’, ‘2011–2012’, and ‘2013–2014’ was 78.4, 79.4, 72.6, and 71.0%, respectively. Overall, 65,973 Korean and 40,617 American respondents participated in KNHANES and NHANES, respectively, during the study period (total *n* = 106,590). Respondents were excluded from analysis if they had been surveyed more than twice in the same year, were aged< 25 or > 65 years (or their age was not documented), or their household income level or educational level was not recorded.

**Fig. 1:**
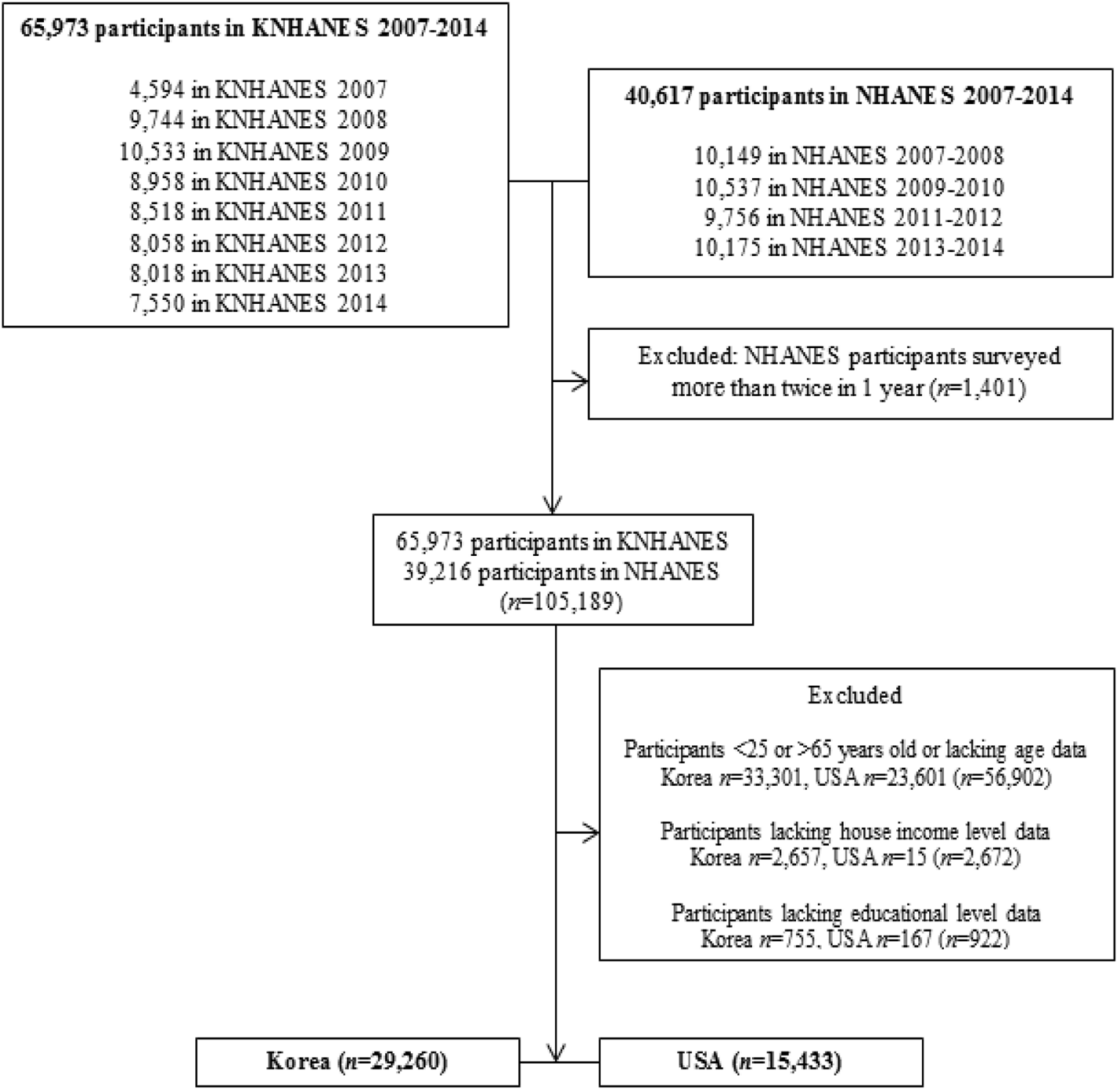
Flowchart of the study population. A: Hypertension B: Diabetes C: Dyslipidemia D: Cardiovascular disease E: Cancer

The final study cohort comprised 44,693 subjects, of whom 29,260 were Korean and 15,443 were American (Fig. 1). Americans were categorized according to ethnicity (e.g., Mexican American, other Hispanic American, non-Hispanic White American, non-Hispanic Black American, and non-Hispanic Asian American). This study was approved by the Institutional Review Board of The Catholic University of Korea (IRB approval number: MC17ZESI0123).

### Study variables

#### Dependent variables

The dependent variable was a “yes” or “no” answer to the question “Have you worked for more than 1 hour during the last week in your current job or business?”

#### Independent variables

The following demographic, socio-economic, and health-related behavior characteristics were recorded. Age was categorized as 25–35, 36–45, 46–55, and 56–65 years. Gender was categorized as male and female. Household total income from each database was classified in terms of quartiles: the bottom 25% were classified as the lower quartile group, followed by the second, third, and upper quartile groups. Education level was categorized as ≤elementary school, middle school, high school, and ≥ college. Current marital status was categorized as never married, married, and widowed/divorced/separated.

Whether the respondent had health insurance was also recorded. In the case of Korea, all Koreans are covered by the national health insurance system. By contrast, the USA has a different health insurance system: medical services are either covered by governmental programs such as Medicare and Medicaid (termed public health insurance) or by private health insurance paid by the employer or the respondent. If medical service costs were covered by any of these sources, the American respondent was deemed to have health insurance. In addition, the American respondents who had private health insurance were classified as ‘yes’ in terms of the ‘Private health insurance’ variable. Koreans can also buy private health insurance: those who did were categorized as ‘yes’ in terms of the ‘Private health insurance’ variable.

Health behaviors factors of study were indicated as Body Mass Index (BMI), smoking status and alcohol consumption. BMI (kg/m^2^) was categorized as underweight (< 18.5), normal weight (18.5–24.9), or overweight (≥ 25). Smoking status was categorized as ‘yes’ if the respondent reported smoking at least 100 cigarettes over the course of their lifetime. Alcohol consumption was categorized as ‘yes’ if the respondent drank more than once a month over their entire life.

Chronic diseases were categorized as being diagnosed with disease by a physician. The main chronic diseases were hypertension, diabetes, dyslipidemia, stroke, myocardial infarction, angina pectoris, and cancer. Respondents were deemed to have cardiovascular disease if they had been diagnosed with stroke, myocardial infarction, or angina pectoris. Respondents were deemed to have cancer if they had been diagnosed with gastric, liver, colon, breast, cervical, or lung cancer and other etc. Thus, the following chronic diseases were examined in this study: hypertension, diabetes mellitus, dyslipidemia, cardiovascular disease, and cancer.

### Statistical analysis

The frequencies of Korean and American respondents with each of the socio-demographic characteristics, health-related behaviors, and chronic diseases were calculated. Data from the two groups were compared using Chi-squared tests.

The ability of chronic disease to independently predict employment status was determined by multiple logistic regression analyses that were adjusted for age, gender, household income level, and education level. These data were expressed as odds ratios (ORs) with 95% confidence intervals (95% CIs).

All analyses were performed using SAS software version 9.4 (SAS Institute, Cary, NC). *P* values of < 0.05 were considered to indicate statistical significance.

## Results

### General characteristics of the study populations

The general characteristics of the study subjects are presented in Table 1. Compared with Americans, Koreans were on average younger (44.1 vs. 45.0 years old, respectively; *P* < 0.001) and more likely to be female (57.7% vs. 51.4%, respectively; *P* < 0.001). The five ethnic groups in the USA did not differ significantly in terms of age or gender. Thus, they were all, including the Asian Americans, older and more likely to be female than the Koreans (all *P* < 0.001).

**Table 1:** Distribution of general characteristics of the study populations by USA and South Korea (*n* = 44,693)

Characteristics	USA						Korea	*P*^c^
	Mexican American	Other Hispanic	Non- Hispanic White	Non- Hispanic Black	Non- Hispanic Asian	Total		
	n(%)	n(%)	n(%)	n(%)	n(%)	n(%)	n(%)	
Gender								
Male	1253(49.8)	764(45.6)	3079(49.5)	1625(48.4)	783(47.1)	7504(48.6)	12,365(42.3)	<.001
Female	1261(50.2)	913(54.4)	3140(50.5)	1735(51.6)	880(52.9)	7929(51.4)	16,895(57.7)	
Age, Mean (SE)						44.1(0.06)	45.0(0.09)	
25–35	677(26.9)	418(24.9)	1610(25.9)	817(24.3)	476(28.6)	3998(25.9)	7480(25.6)	<.001
36–45	706(28.1)	402(24.0)	1617(26.0)	728(21.7)	464(27.9)	3917(25.4)	8903(30.4)	
46–55	572(22.8)	401(23.9)	1565(25.2)	857(25.5)	392(23.6)	3787(24.5)	8375(28.6)	
56–65	559(22.2)	456(27.2)	1427(22.9)	958(28.5)	331(19.9)	3731(24.2)	4502(15.4)	
BMI^a^								
Under weight	475(18.9)	308(18.4)	1121(18.0)	650(19.3)	314(18.9)	2868(18.6)	1308 (4.5)	<.001
Normal	678(27.0)	476(28.4)	1708(27.5)	905(26.9)	485(29.2)	4252(27.6)	18,589(63.5)	
Over weight	1142(45.4)	758(45.2)	2892(46.5)	1520(45.3)	717(43.1)	7029(45.5)	9363(32.0)	
Smoking								
Yes	708(28.2)	456(27.2)	1713(27.5)	909(27.0)	360(21.7)	4146(26.9)	12,052(41.2)	<.001
No	1806(71.8)	1221(72.8)	4506(72.5)	2451(73.0)	1303(78.3)	11,287(73.1)	17,208(58.8)	
Drinking								
Yes	1287(51.2)	811(48.4)	2974(47.8)	1474(43.9)	449(27.0)	6995(45.3)	16,818(57.5)	<.001
No	1227(48.8)	866(51.6)	3245(52.2)	1886(56.1)	1214(73.0)	8438(54.7)	12,442(42.5)	
House income level								
Upper quartile	506(20.1)	466(27.8)	2250(36.2)	825(24.5)	744(44.7)	4791(31.0)	9565(32.7)	<.001
Second quartile	941(37.5)	567(33.8)	1784(28.7)	1158(34.5)	496(29.8)	4946(32.1)	9242(31.6)	
Third quartile	617(24.5)	316(18.8)	1025(16.5)	659(19.6)	227(13.7)	2844(18.4)	7406(25.3)	
Lower quartile	450(17.9)	328(19.6)	1160(18.6)	718(21.4)	196(11.8)	2852(18.5)	3047(10.4)	
Education level								
≤ Elementary school	797(31.7)	315(18.8)	167(2.7)	96(2.9)	109(6.5)	1484(9.6)	2087(7.1)	<.001
Middle school	525(20.9)	292(17.4)	753(12.1)	633(18.8)	126(7.6)	2329(15.1)	3108(10.6)	
High school	491(19.5)	343(20.4)	1441(23.2)	899(26.8)	256(15.4)	3430(22.2)	7449(25.5)	
≥ College	701(27.9)	727(43.4)	3858(62.0)	1732(51.5)	1172(70.5)	8190(53.1)	16,616(56.8)	
Marital status								
Never married	270(10.7)	239(14.3)	874(14.1)	940(28.0)	252(15.1)	2575(16.7)	3565(12.2)	<.001
Married	1822(72.5)	1079(64.3)	4138(66.5)	1592(47.4)	1227(73.8)	9858(63.9)	23,460(80.2)	
Divorced/separated/ widowed	422(16.8)	359(21.4)	1207(19.4)	828(24.6)	184(11.1)	3000(19.4)	2235(7.6)	
Public health insurance								
Yes	1254(49.9)	1099(65.5)	4918(79.1)	2532(75.4)	1297(78.0)	11,100(71.9)	29,135(99.6)	<.001
No	1260(50.1)	578(34.5)	1301(20.9)	828(24.6)	366(22.0)	4333(28.1)	125(0.4)	
Private health insurance								
Yes	902(35.9)	742(44.3)	3871(62.2)	1645(49.0)	1003(60.3)	8163(52.9)	24,259(82.9)	<.001
No	1612(64.1)	935(55.7)	2348(37.8)	1715(51.0)	660(39.7)	7270(47.1)	5001(17.1)	
Hypertension								
Yes	569(22.6)	404(24.1)	1421(22.9)	715(21.3)	264(15.9)	3373(21.9)	3770(12.9)	<.001
No	1945(77.4)	1273(75.9)	4798(77.2)	2645(78.7)	1399(84.1)	12,060(78.1)	25,490(87.1)	
Diabetes								
Yes	163(6.5)	128(7.6)	450(7.2)	258(7.7)	141(8.5)	1140(7.4)	1501(5.1)	<.001
No	2351(93.5)	1549(92.4)	5769(92.8)	3102(92.3)	1522(91.5)	14,293(92.6)	27,759(94.9)	
Dyslipidemia								
Yes	523(20.8)	333(19.9)	1273(20.5)	656(19.5)	235(14.1)	3020(19.6)	2378(8.1)	<.001
No	1991(79.2)	1344(80.1)	4946(79.5)	2704(80.5)	1428(85.9)	12,413(80.4)	26,882(91.9)	
Cardiovascular disease^b^								
Yes	119(4.7)	79(4.7)	328(5.3)	175(5.2)	84(5.1)	785(5.0)	648(2.2)	<.001
No	2395(95.3)	1598(95.3)	5891(94.7)	3185(94.8)	1579(94.9)	14,648(95.0)	28,612(97.8)	
Cancer								
Yes	158(6.3)	89(5.3)	355(5.7)	186(5.5)	102(6.1)	890(5.8)	626(2.1)	<.001
No	2356(93.7)	1588(94.7)	5664(94.3)	3174(94.5)	1561(93.9)	14,543(94.2)	28,634(97.9)	
Employment status								
Employed	936(37.2)	596(35.5)	2206(35.5)	1123(33.4)	406(24.4)	5267(34.1)	19,754(67.5)	<.001
Unemployed	1578(62.8)	1081(64.5)	4013(64.5)	2237(66.6)	1257(75.6)	10,166(65.9)	9506(32.5)	
Total	2514(100.0)	1677(100.0)	6219(100.0)	3360(100.0)	1663(100.0)	15,433(100.0)	29,260(100.0)	

*Abbreviations*: *BMI* body mass index; *SE* standard error

^a^BMI (kg/m^2^): underweight(< 18.5); normal(18.5–24.9); overweight(≥25)

^b^Cardiovascular disease includes stroke, myocardial infarction and angina pectoris

^c^The *p*-value calculated by comparing between Korea and the total of United States

Compared with the Americans, the Koreans were more likely to have a normal weight (63.5% vs. 27.6%, respectively; *P* < 0.001) and less likely to be overweight (32.0% vs. 45.5%, respectively; *P* < 0.001). The American ethnic groups did not differ significantly in terms of BMI, and all (including Asian Americans) were more likely to be overweight than the Koreans (all *P* < 0.001) (Table 1).

Koreans were more likely to smoke (41.2% vs. 26.9%, respectively; *P* < 0.001) and drink (57.5% vs. 45.3%, respectively; *P* < 0.001) than the Americans. In terms of American ethnic groups, Mexican Americans had higher rates of smoking (28.2%) and Asian Americans had lower rates of smoking (21.7%) than the other American races (27.2–27.5%, all *P* < 0.001). This trend was also observed for drinking: Mexican Americans had higher rates of drinking (51.2%) and Asian Americans had lower rates of drinking (27.0%) than other Americans races (43.9–48.4%, all *P* < 0.001) (Table 1).

Americans were more likely than Koreans to be in the lower quartile of household income (10.4% vs. 18.5%, respectively) and more likely to be in the third quartile (25.3% vs. 18.4%, respectively; *P* < 0.001). Regarding the American ethnic groups, Black Americans were most likely to be in the lowest quartile of household income (21.4%) while Asian Americans were less likely to be in this quartile (11.8%) than the other groups (17.9–19.6%, all *P* < 0.001). Mexican Americans were more likely (24.5%), while Asian Americans were less likely (13.7%), to be in the third quartile of household income than the other groups (16.5–19.6%, all *P* < 0.001).

In terms of education level, Koreans were more likely to be college graduates than Americans (56.8% vs. 53.1%, respectively) and less likely to have elementary education (7.1% vs. 9.6%, respectively; *P* < 0.001). Of the American ethnic groups, Mexican Americans were less likely to be college graduates (27.9%) whereas Asian Americans were most likely to have graduated from college (70.5%) than the other groups (43.4–62.0%, *P* < 0.001) (Table 1).

Koreans were more likely to be married than the Americans (80.2% vs. 63.9%, respectively; *P* < 0.001). Asian Americans and Mexican Americans also had higher rates of marriage (73.8 and 72.5%, respectively) than other ethnic groups in the USA (47.4–66.5%, *P* < 0.001) (Table 1).

Koreans were more likely to have public health insurance than Americans (99.6% vs. 71.9%, respectively; *P* < 0.001). The Koreans were also more likely than Americans to have private health insurance (82.9% vs. 52.9%, respectively; *P* < 0.001). In terms of American ethnic groups, White Americans had the highest rates of public health insurance (79.1%) and private health insurance (62.2%) while Mexican Americans had the lowest rates of these insurances (49.9 and 35.9%, respectively). Asian Americans resembled the White Americans in terms of public and private health insurance rates (78.0 and 60.3%, respectively) (all *P* < 0.001).

In terms of chronic disease rates, compared with the Americans, the Koreans had lower rates of hypertension (21.9% vs. 12.9%, respectively; *P* < 0.001), and also lower rates of diabetes mellitus (5.1% vs. 7.4%, respectively; *P* < 0.001), dyslipidemia (8.1% vs. 19.6%, respectively; *P* < 0.001), cardiovascular disease (2.2% vs. 5.0%, respectively; *P* < 0.001), and cancer (2.1% vs. 5.8%, respectively; *P* < 0.001). The different ethnic groups in the USA had similar rates of hypertension (21.3–24.1%), cardiovascular disease (4.7–5.3%), and cancer (5.3–6.3%). However, compared with the other races, Asian Americans were significantly more likely to have diabetes mellitus (8.5% vs. 6.5–7.7%, *P* < 0.001) and significantly less likely to have hypertension (15.9% vs. 21.3–24.1%, *P* < 0.001) and dyslipidemia (14.1% vs. 19.5–20.8%, *P* < 0.001) (Table 1).

Koreans were less likely to be unemployed than Americans (32.5% vs. 65.9%, respectively; *P* < 0.001). Thus, American respondents were twice as likely to be unemployed than employed, while the reverse was true for Korean respondents (Table 1).

### Comparing Koreans and Americans in terms of the association between chronic disease and employment status

**Fig. 2:**
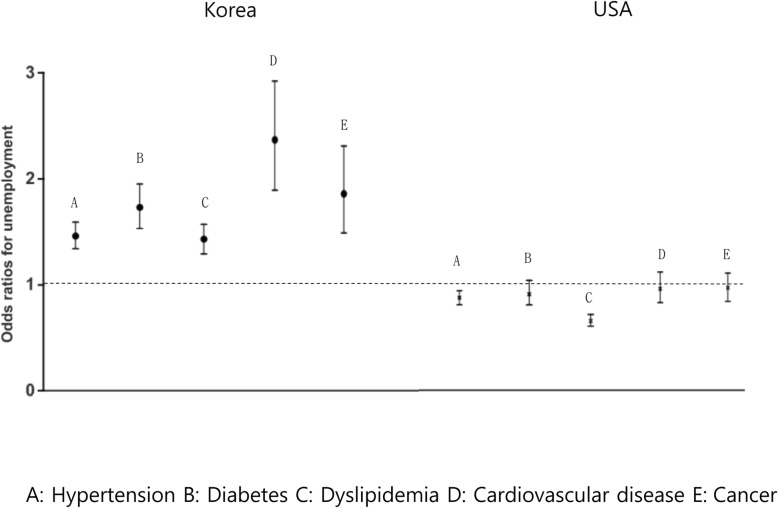
Association between chronic disease and unemployment status according to nationality. **a**: Hypertension **b**: Diabetes **c**: Dyslipidemia **d**: Cardiovascular disease **e**: Cancer

To assess the association between chronic disease and employment in Korea and the USA, multivariate logistic regression analysis was performed after adjusting for age, gender, total household income, and education level (Fig. 2).

Thus, Koreans with hypertension were two times more likely to be unemployed (OR = 1.46, 95% CI = 1.34–1.59) than hypertensive Americans (OR = 0.87, 95% CI = 0.80–0.94), while Koreans with diabetes mellitus were twice as likely to be unemployed (OR = 1.80, 95% CI = 1.53–1.95) than diabetic Americans (OR = 0.90, 95% CI = 0.80–1.03). Moreover, Koreans with dyslipidemia were twice as likely to be unemployed (OR = 1.43, 95% CI = 1.29–1.57) than Americans with dyslipidemia (OR = 0.65, 95% CI = 0.60–0.71).

Similarly, Koreans diagnosed with cardiovascular disease were 2.5-fold more likely to be unemployed (OR = 2.47, 95% CI = 2.06–2.95) than Americans with cardiovascular disease (OR = 0.95, 95% CI = 0.82–1.11). Moreover, Koreans with cancer were twice as likely to be unemployed (OR = 2.03, 95% CI = 1.71–2.42) than Americans with cancer (OR = 1.00, 95% CI = 0.82–1.23).

Thus, Korean respondents who had been diagnosed with a chronic disease had higher rates of unemployment than their healthy counterparts. By contrast, in the American respondents, a chronic disease tended to have no effect on or even decrease the risk of unemployment. Thus, in general, the Korean respondents with a chronic disease were 2–3 times more likely to be unemployed than the American respondents with the same chronic disease.

Results of multiple logistic regression analyses after adjusting for age, gender, level of total household income, and education level are shown and conducted a separate analysis for each disease to assess the unemployment status. Data are expressed as the odds ratio (OR) with 95% confidence intervals.

### Comparison of American ethnic groups in terms of the association between chronic disease and employment status

**Fig. 3:**
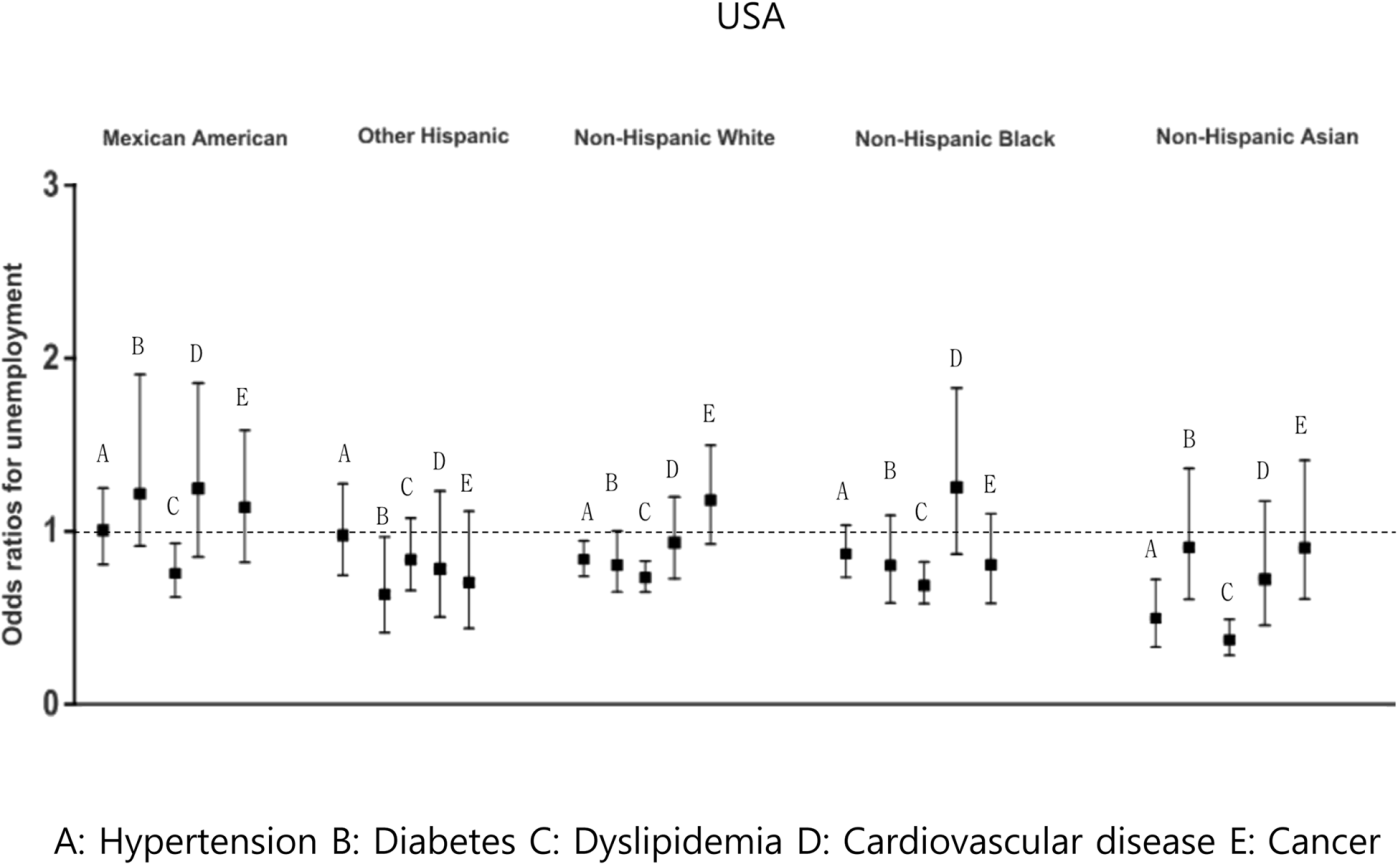
Association between chronic disease and unemployment status in Americans. **a**: Hypertension **b**: Diabetes **c**: Dyslipidemia **d**: Cardiovascular disease **e**: Cancer

To assess the association between chronic disease and employment in the various ethnic groups in the USA (especially the Asian Americans, since they can serve as a genetic control for the Koreans to some extent), similar adjusted multivariate logistic regression analyses were performed (Fig. 3).

There were so few Asian Americans who had been diagnosed with hypertension that their OR is likely to be meaningless; therefore, these odds are not shown in Fig. 3. Hypertension tended to decrease the risk of unemployment in the other American ethnic groups (ORs ranged from 0.5 to 0.6). The Mexican Americans who were diagnosed with diabetes mellitus were more likely to be unemployed than the non-diabetic Mexican Americans (OR = 1.21, 95% CI = 0.87–1.71). This trend was not observed in the other ethnic groups, including the Asian Americans (ORs ranged from 0.5 to 0.9). The Asian Americans who had dyslipidemia were less likely to be unemployed than the Asian Americans without dyslipidemia (OR = 0.36, 95% CI = 0.27–0.48). The other races also showed this protective effect, but it was not as marked as in the Asian Americans (ORs ranged from 0.6 to 0.8).

Mexican Americans who were diagnosed with cardiovascular disease were more likely to be unemployed than the Mexican Americans without this disease (OR = 1.13, 95% CI = 0.77–1.68). This was also observed for the Black Americans (OR = 1.24, 95% CI = 0.89–1.76) but not the other races, including the Asian Americans (ORs ranged from 0.7 to 1.0). The White Americans who were diagnosed with cancer had a higher risk of unemployment than the White Americans without cancer (OR = 1.17, 95% CI = 0.93–1.47). This was also observed for the Mexican Americans (OR = 1.05, 95% CI = 0.75–1.47) but not the other races, including the Asian Americans (ORs ranged from 0.7 to 0.9).

Thus, in Asian Americans, dyslipidemia decreased the risk of unemployment but the other diseases had no or a slightly protective effect on Asian American employment status. In the other groups, cancer increased the risk of unemployment in Whites and Blacks; cardiovascular disease reduced employment in Mexican and Black Americans; and diabetes associated with less employment in Mexican Americans. The other diseases had little effect on the employment rates of the other groups. It is notable that these chronic diseases had much greater deleterious effects on unemployment in Koreans (ORs ranged from 1.17 to 2.47) than in any of the American ethnic groups (ORs ranged from 0.36 to 1.24), including the Asian Americans (ORs ranged from 0.36 to 0.9).

### Comparing Koreans and Americans in terms of the association between chronic disease and employment status according to income level

**Fig. 4:**
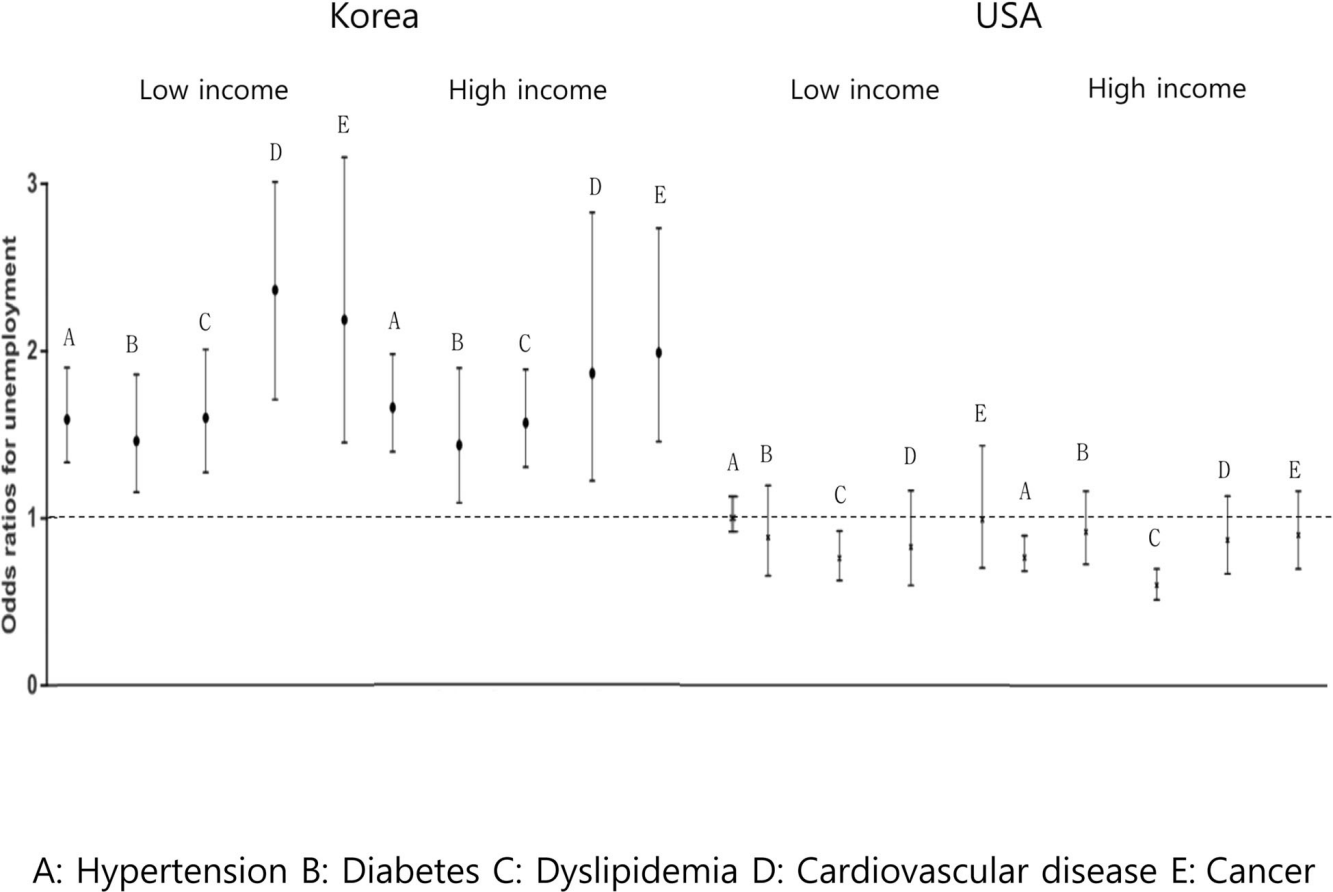
Association between chronic disease and unemployment status of Koreans and Americans according to income level

To assess the association between chronic disease and employment in Korea and the USA according to income level, we performed multivariate logistic regression analysis after adjusting age, gender, and education level (Fig. 4).

Korean respondents with a low level of household income who had been diagnosed with a chronic disease had slightly higher rates of unemployment than their high income counterparts. Koreans with a low income who were diagnosed with cardiovascular disease were more likely to be unemployed (OR = 2.38, 95% CI = 1.72–3.03) than those with a high income (OR = 1.82, 95% CI = 1.19–2.76). Similarly, Koreans with a low income were more likely to be unemployed (OR = 2.20, 95% CI = 1.46–3.18) than those with a high income (OR = 1.94, 95% CI = 1.42–2.67). By contrast, income level had no effect on the risk of Americans with a chronic disease being unemployed.

Thus, in general, Korean respondents with a chronic disease and a low level of household income were more likely to be unemployed.

## Discussion

People with chronic diseases find it difficult to maintain their job and participate in the community [7]. This study asked whether the impact of chronic diseases on employment is similar in Korea and the USA. It showed that, in fact, although Korean respondents were more likely in general to be employed than American respondents, they were relatively less likely to be employed if they had a chronic disease.

The differences between Korea and the USA in terms of the relationship between chronic disease and unemployment status may be due to health insurance coverage, maintenance of coverage after voluntary unemployment, utilization of rehabilitation programs, cultural approaches, etc.

First, the scope of health insurance coverage and state benefits for unemployed people is different from country to country. The majority of Korean respondents were more likely to have public or private health insurance than Americans. This reflects the fact that Korea has universal health insurance and the USA does not. The healthcare security of Koreans is covered by the national health insurance system, which is supported by the law and requires compulsory subscription. Thus, Koreans who become unemployed are transferred to a local subscriber, which maintains their health insurance status [16]. By contrast, in the USA, even basic guarantees are difficult to obtain, and most Americans only receive health insurance when they make a contract with a company [17]. This may explain why Americans with chronic diseases are more likely to be employed than Koreans with the same diseases.

Second, chronic diseases such as cardiovascular disease and cancer affect the ability of the individual to retain a job, making it more likely that they will be unemployed [18, 19]. Cancer patients generally lose their job within a year of diagnosis because of the need to focus on their treatment [16, 20]. Koreans with cancer were twice as likely to be unemployed than Americans with cancer. In Korea, young cancer patients return to work more frequently than older patients; this may reflect the fact that older patients are more likely to retire from their job if they develop a chronic disease [21]. Koreans are covered by basic health insurance no matter what their retirement status; therefore, they may have less motivation to keep working when they have a severe illness that requires protracted treatment. However, most Americans may have to bear the costs of their own treatment, especially after a certain period of time; this motivates the individual to continue working. Federal laws in the USA (including COBRA [Consolidated Omnibus Budget Reconciliation Act] and similar laws) do allow people to continue their health insurance coverage for an 18-month maximum period of time after they leave contractual employment [10, 22], meaning that they may not be regarded as unemployed. Thus, Americans with chronic diseases can maintain employment status for a period time, even if they have a disease.

Third, utilization of rehabilitation programs may affect the employment status of people with cardiovascular disease and cancer. Americans with cardiovascular disease are more likely to undergo cardiac rehabilitation programs, which can prevent death or serious disabilities [23, 24] and can accelerate the patient’s return to work [25, 26]. However, there might be obstacles to utilization of rehabilitation program for Koreans with cardiovascular disease due to limited coverage by the insurance system; also, limited sources [27, 28] can prevent patients with cardiovascular disease from returning to work. Therefore, the possibility of return-to-work might be less in Korea than US.

Fourth, the workplace environment and cultural attitudes to those with a disability may influence the employment status of those with cardiovascular disease and cancer. Koreans with cancer have experienced discrimination in the workplace due to disabilities, which makes it less likely that they will seek out employment [29]. There is a similar phenomenon in USA, but monitoring systems prohibit discrimination against people with disabilities. In the USA, any discrimination should be reported to the Equal Employment Opportunity Commission (EEOC) and, if justified, an order to prohibit the violation in the workplace is issued [30, 31]. Thus, the system in the USA makes it easier for people with chronic diseases to remain in work.

Socio-economic factors such as income level are predictors of unemployment [16]. However, our results revealed no difference in unemployment rates among those with chronic diseases after stratifying according to income (Fig. 4). Figure 4 shows no association between ethnicity (as a surrogate of SES) in the USA and unemployment. The issue of SES might not be potential confounder with respect to the association between unemployment and prevalent chronic diseases.

Several limitations of this study must be considered in the interpretation of findings. First, the study design was a cross-sectional study, so the causal relationship between chronic illness and employment status could not be assessed. Second, a potential information bias due to using KNHANES and NHANES should be considered. The exact reasons of leaving their job such as leave, holiday, and education were not assessed in the questionnaires. In addition, further prospective study on the possibility of working part-time might, for example, differ between the two countries in terms of possibility (part-time work is available, employees with chronic health conditions can work reduced hours) and feasibility (the income from their work is sufficient to live on or can be made up by e.g. government welfare payments) should be followed.

Despite its limitations, this study has several strengths. First, it used two large survey databases comprising 44,693 respondents and thus made a comprehensive assessment of the link between diagnosed chronic disease and economic activity. Second, the study focused on the effect of not just one chronic disease but five on unemployment rate. Third, the study was able to compare two countries directly because the data capture systems were extremely similar. Finally, the study not only compared two countries; it also compared various ethnic groups in the USA. This allowed us to show that chronic disease had a consistently smaller effect on employment status in Americans than in Korea.

## Conclusions

In conclusion, chronic disease had a significant impact on economic activity in Korea and a smaller impact on economic activity in the USA. This difference may be related to the different health insurance systems and cultural approaches to people with diseases in the two countries. Thus, although chronic disease can now be managed effectively by existing health care systems, it remains a problem that can undermine the socio-economic development of a nation significantly. To limit this, it is important to explore factors that limit the economic participation of people with chronic diseases and to identify social policies that will overcome these factors. As such, further research should identify other variables that explain the direct or indirect impact of chronic diseases on employment.

## Data Availability

Not applicable.

## Abbreviations

NCDs: Non-Communicable Diseases
USA: United States of America
NHANES: National Health and Nutrition Examination Survey
IRB: Institutional Review Board
BMI: Body Mass Index
SE: Standard Error
COBRA: Consolidated Omnibus Budget Reconciliation Act
EEOC: Equal Employment Opportunity Commission
SES: Socioeconomic status
